# Role of *MUC1* rs4072037 polymorphism and serum KL-6 levels in patients with antisynthetase syndrome

**DOI:** 10.1038/s41598-021-01992-y

**Published:** 2021-11-19

**Authors:** Sara Remuzgo-Martínez, Belén Atienza-Mateo, J. Gonzalo Ocejo-Vinyals, Fernanda Genre, Verónica Pulito-Cueto, Víctor M. Mora-Cuesta, David Iturbe-Fernández, Leticia Lera-Gómez, Raquel Pérez-Fernández, Diana Prieto-Peña, Juan Irure, Fredeswinda Romero-Bueno, Olga Sanchez-Pernaute, Rodrigo Alonso-Moralejo, Laura Nuño, Gema Bonilla, Esther F. Vicente-Rabaneda, Ignacio Grafia, Sergio Prieto-González, Javier Narvaez, Ernesto Trallero-Araguas, Albert Selva-O’Callaghan, Spanish Biomarkers of Antisynthetase Syndrome Consortium, Norberto Ortego-Centeno, Nair Pérez-Gómez, Antonio Mera, Julia Martínez-Barrio, Clara Moriano, Elvira Díez, Jaime Calvo-Alén, Alejandro Balsa, Spanish Biomarkers of Interstitial Lung Disease Consortium, María Piedad Ussetti, Rosalía Laporta, Cristina Berastegui, Amparo Solé, Oreste Gualillo, Lorenzo Cavagna, José M. Cifrián, Elisabetta A. Renzoni, Santos Castañeda, Raquel López-Mejías, Miguel A. González-Gay

**Affiliations:** 1 Research Group on Genetic Epidemiology and Atherosclerosis in Systemic Diseases and in Metabolic Diseases of the Musculoskeletal System, IDIVAL, Avenida Cardenal Herrera Oria s/n, Lab. 201/202, 39011 Santander, Spain; 2 López Albo’ Post-Residency Programme, Hospital Universitario Marqués de Valdecilla, Santander, Spain; 3 Rheumatology Department, Hospital Universitario Marqués de Valdecilla, Santander, Spain; 4 Department of Immunology, Hospital Universitario Marqués de Valdecilla, Santander, Spain; 5 Pneumology Department, Hospital Universitario Marqués de Valdecilla, Santander, Spain; 6 Rheumatology Department, Hospital Universitario Fundación Jiménez Díaz, Madrid, Spain; 7 Lung Transplant Unit, Hospital Universitario 12 de Octubre, Madrid, Spain; 8 Rheumatology Department, Hospital Universitario La Paz, Madrid, Spain; 9 Rheumatology Department, Hospital de la Princesa, IIS-Princesa, Madrid, Spain; 10 Department of Autoimmune Diseases, Hospital Clínico de Barcelona, Universidad de Barcelona, Barcelona, Spain; 11 Rheumatology Department, Hospital Universitario de Bellvitge, Barcelona, Spain; 12 Rheumatology Unit, Hospital Universitario Vall d’Hebron, Barcelona, Spain; 13 Systemic Autoimmune Diseases Unit, Hospital Universitario Vall d’Hebron, Barcelona, Spain; 14 SERGAS (Servizo Galego de Saude) and IDIS (Instituto de Investigación Sanitaria de Santiago), NEIRID Lab (Neuroendocrine Interactions in Rheumatology and Inflammatory Diseases), Research Laboratory 9, Hospital Clínico Universitario de Santiago, Santiago de Compostela, Spain; 15 Division of Rheumatology, University and IRCCS Policlinico S. Matteo Foundation, Pavia, Italy; 16 School of Medicine, Universidad de Cantabria, Santander, Spain; 17 Interstitial Lung Disease Unit, Royal Brompton Hospital, Imperial College London, London, UK; 18 Cardiovascular Pathophysiology and Genomics Research Unit, School of Physiology, Faculty of Health Sciences, University of the Witwatersrand, Johannesburg, South Africa; 19 Department of Medicine, Universidad de Granada, Granada, Spain; 20 Division of Rheumatology, Instituto de Investigación Sanitaria-Hospital Clínico Universitario de Santiago, Santiago de Compostela, A Coruña Spain; 21 Department of Rheumatology, Hospital General Universitario Gregorio-Marañón, Madrid, Spain; 22 Division of Rheumatology, Complejo Asistencial Universitario de León, León, Spain; 23 Rheumatology Division, Hospital Universitario Araba, Vitoria/Gasteiz, Álava Spain; 24 Pneumology Department, Hospital Universitario Puerta de Hierro, Majadahonda, Spain; 25 Pneumology Department, Hospital Universitario Vall d’Hebron, Universidad Autónoma de Barcelona, Barcelona, Spain; 26 Lung Transplant and Cystic Fibrosis Unit, Hospital Universitario y Politécnico La Fe, Valencia, Spain

**Keywords:** Biomarkers, Idiopathic inflammatory myopathies

## Abstract

Mucin 1/Krebs von den Lungen-6 (KL-6) is proposed as a serum biomarker of several interstitial lung diseases (ILDs), including connective tissue disorders associated with ILD. However, it has not been studied in a large cohort of Caucasian antisynthetase syndrome (ASSD) patients. Consequently, we assessed the role of *MUC1* rs4072037 and serum KL-6 levels as a potential biomarker of ASSD susceptibility and for the differential diagnosis between patients with ILD associated with ASSD (ASSD-ILD +) and idiopathic pulmonary fibrosis (IPF). 168 ASSD patients (149 ASSD-ILD +), 174 IPF patients and 523 healthy controls were genotyped for *MUC1* rs4072037 T > C. Serum KL-6 levels were determined in a subgroup of individuals. A significant increase of *MUC1* rs4072037 CC genotype and C allele frequencies was observed in ASSD patients compared to healthy controls. Likewise, *MUC1* rs4072037 TC and CC genotypes and C allele frequencies were significantly different between ASSD-ILD+ and IPF patients. Additionally, serum KL-6 levels were significantly higher in ASSD patients compared to healthy controls. Nevertheless, no differences in serum KL-6 levels were found between ASSD-ILD+ and IPF patients. Our results suggest that the presence of *MUC1* rs4072037 C allele increases the risk of ASSD and it could be a useful genetic biomarker for the differential diagnosis between ASSD-ILD+ and IPF patients.

## Introduction

Antisynthetase syndrome (ASSD) is a connective tissue disease (CTD) within the group of idiopathic inflammatory myopathies (IIMs), mainly characterized by the clinical triad of arthritis, myositis and interstitial lung disease (ILD). In this regard, ILD is the most common and serious internal organ involvement of ASSD patients^1–5^. However, the pathogenesis of ASSD is still unclear and its diagnosis is somewhat delayed when typical features are absent. Consequently, further research in this context is needed.

Mucin 1, better known as Krebs von den Lungen-6 (KL-6), is one of the key transmembrane mucins in the lung, mainly implicated in processes of cellular proliferation, growth and apoptosis, with increased expression in injured or regenerating epithelial cells^6–8^. Its role as a serum biomarker has been widely studied in several ILDs, including idiopathic pulmonary fibrosis (IPF) and CTDs associated with ILD (CTD-ILD). In fact, it is considered as a useful marker of epithelial lung damage and a predictor of fibrotic progression in these diseases^6–17^. In addition, the functional polymorphism rs4072037, located at nucleotide position 568 in the exon 2 of *MUC1* gene, affects serum KL-6 levels^9,14,18–21^. An association between *MUC1* rs4072037 and diverse pathologies has also been reported^21–24^.

There are several studies focused on the association of KL-6 with other IIMs, mainly polymyositis (PM) and dermatomyositis (DM), that indicate a relevant role of this marker in the development and progression of ILD in this context^12,16,17,25–30^. However, to the best of our knowledge, the effect of *MUC1* rs4072037 and serum KL-6 levels in a large cohort of Caucasian patients with ASSD remains to be elucidated. Therefore, in this study we evaluated the role of mucin 1/KL-6, at the genetic and serological level, as a potential biomarker of ASSD. For this purpose, we aimed to explore its influence on the susceptibility to ASSD and its possible use for the differential diagnosis between ASSD patients with ILD (ASSD-ILD +) and patients with IPF.

## Methods

### Patients and controls

A total of 168 unrelated Spanish patients of European ancestry diagnosed with ASSD (149 ASSD-ILD +) were enrolled in this cross-sectional study from the following Spanish hospitals: Hospital Universitario Marqués de Valdecilla (Santander), Hospital Clínico Universitario de Santiago (Santiago de Compostela), Hospital Universitario San Cecilio (Granada), Hospital Universitario Araba (Vitoria), Complejo Asistencial Universitario de León (León), Hospital Universitario Vall d´Hebron, Hospital Clínico de Barcelona and Hospital Universitario de Bellvitge (Barcelona), Hospital Universitario de la Princesa, Hospital Universitario La Paz, Hospital Universitario Fundación Jiménez Díaz, and Hospital General Universitario Gregorio Marañón (Madrid). As we originally defined^31,32^, these patients were recruited if they had an antisynthetase antibody positive in at least two determinations along with one or more findings of arthritis, myositis and/or ILD^2–4,33^. ILD was defined instrumentally by forced vital capacity (FVC) ≤ 80%, forced expiratory volume in one second/FVC ≥ 70%] and/or diffusing capacity of the lung for carbon monoxide (DLCO) < 80% and interstitial changes on chest high-resolution computed tomography^2–4^. Antisynthetase autoantibodies were detected by the myositis immunoblot kit ‘Euroline Autoimmune Inflammatory Myopathies 16 Ag kit’ (Euroimmun, Luebeck, Germany) or ENA (extractable nuclear antigen) screen tests for anti Jo-1 antibodies, in some cases, as we described^32^. The occurrence of fever, Raynaud’s phenomenon and mechanic’s hands was also recorded^34,35^. Patients with other IIMs were excluded. Demographic and clinical characteristics of the whole cohort of ASSD patients included in this study have been previously reported^32^.

In addition, 174 unrelated Spanish patients of European ancestry who fulfilled the American Thoracic Society/European Respiratory Society classification and diagnosis criteria for IPF^36,37^ were recruited from Hospital Universitario Marqués de Valdecilla (Santander), Hospital Universitario Vall d´Hebrón (Barcelona), Hospital Universitario y Politécnico de la Fe (Valencia), and Hospital Universitario 12 de Octubre and Hospital Universitario Puerta de Hierro (Madrid).

The main demographic and baseline lung function information of ASSD-ILD+ and IPF patients included in this study is described in Supplementary table 1.

Moreover, a set of 523 ethnically‐matched healthy controls (44.3% men/55.7% women), without history of any autoimmune or pulmonary disease, constituted by blood donors from Hospital Universitario Marqués de Valdecilla (Santander) and National DNA Bank Repository (Salamanca), was also included in this study. Their mean age ± standard deviation (SD) at the time of the study was 52.8 ± 11.1 years.

For experiments involving humans and the use of human blood samples, all the methods were carried out in accordance with the approved guidelines and regulations, according to the Declaration of Helsinki. All experimental protocols were approved by the local Ethics Committee of each participant hospital: Hospital Universitario Marqués de Valdecilla (Santander), Hospital Clínico Universitario de Santiago (Santiago de Compostela), Hospital Universitario San Cecilio (Granada), Hospital Universitario Araba (Vitoria), Complejo Asistencial Universitario de León (León), Hospital Universitario y Politécnico de la Fe (Valencia), Hospital Universitario Vall d´Hebrón, Hospital Clínico de Barcelona and Hospital Universitario de Bellvitge (Barcelona), Hospital Universitario de la Princesa, Hospital Universitario La Paz, Hospital Universitario Fundación Jiménez Díaz, Hospital General Universitario Gregorio Marañón, Hospital Universitario 12 de Octubre and Hospital Universitario Puerta de Hierro (Madrid). All individuals signed an informed written consent to be included in the study.

### *MUC1* rs4072037 genotyping

Genomic DNA from all the patients and healthy controls was extracted from peripheral blood using the REALPURE “SSS” kit (RBME04, REAL, Durviz S.L., Valencia, Spain). All the individuals were genotyped for *MUC1* rs4072037 T > C by a TaqMan assay (C__27532642_10) in a QuantStudio™ 7 Flex real-time polymerase chain reaction system (Applied Biosystems, Foster City, CA, USA). Negative controls and duplicate samples were included to check the accuracy of the genotyping.

### Determination of serum KL-6 levels

Serum KL-6 levels were measured in a total of 213 individuals (74 ASSD patients (66 ASSD-ILD +), 80 IPF patients and 59 healthy controls) by using a chemiluminescent enzyme immunoassay (Lumipulse G KL-6, Fujirebio Iberia SLU) and analyzed in the Fujirebio Lumipulse G600 II instrument.

### Statistical analysis

Data were reported as number of individuals (n), percentage (%), and mean ± SD, as appropriate.

All genotype data were checked for deviation from Hardy–Weinberg equilibrium (HWE). Both genotype and allele frequencies of *MUC1* rs4072037 were calculated and compared between ASSD patients and healthy controls as well as between ASSD-ILD+ patients and IPF patients by chi-square test. Strength of associations was estimated by logistic regression using odds ratios (OR) and 95% confidence intervals (CI). Results were also adjusted for age and sex by logistic regression.

Since ILD is the most serious complication of ASSD and anti Jo-1 the most frequent antisynthetase antibody^1–3,5^, genetic differences between ASSD patients stratified according to presence/absence of ILD or anti Jo-1 antibodies positive/negative were assessed. The strength of associations was estimated by logistic regression using OR and 95% CI. Results were also adjusted for age and sex by logistic regression.

Differences in serum levels of KL-6 between two study groups were calculated by Student’s t-test and further adjusted for age, sex, and smoking history using ANCOVA.

Given the remarkable difference in men/women ratio between ASSD-ILD+ patients and IPF patients, genetic and serum data were also analyzed stratifying the population according to sex.

The influence of *MUC1* rs4072037 genotypes and alleles on serum KL-6 levels was tested using linear regression.

In all cases, p-values ≤ 0.05 were considered as statistically significant. All statistical analyses were performed with STATA statistical software 12/SE (Stata Corp., College Station, TX, USA).

## Results

### Effect of *MUC1* rs4072037 in patients with ASSD

We confirmed that the rs4072037 genotype distribution was in HWE (p > 0.05). The rate of genotyping success was 100%. We also found that in our study the genotype and allele frequencies of *MUC1* rs4072037 in healthy controls were similar to the data of the 1000 Genomes Project for Europeans.

We disclosed a statistically significant increase of *MUC1* rs4072037 CC genotype and C allele frequencies in the whole cohort of patients with ASSD when compared to healthy controls (Table 1). This increase remained marginally significant when adjusted for age and sex (Table 1).

**Table 1:** Genotype and allele frequencies of *MUC1* rs4072037 in ASSD patients and healthy controls.

	ASSD % (n)	Healthy controls % (n)	*p*	OR [95% CI]	p*	OR [95% CI]*
**Genotypes**						
TT	20.2 (34)	27.2 (142)	–	Ref.	–	Ref.
TC	50.6 (85)	49.3 (258)	0.16	1.38 [0.88–2.15]	0.16	1.40 [0.88–2.24]
CC	29.2 (49)	23.5 (123)	**0.05**	1.66 [1.01–2.74]	0.08	1.60 [0.95–2.71]
**Alleles**						
T	45.5 (153)	51.8 (542)	–	Ref.	–	Ref.
C	54.5 (183)	48.2 (504)	**0.05**	1.29 [1.01–1.65]	0.08	1.26 [0.97–1.63]

ASSD: antisynthetase syndrome; CI: confidence interval; OR: odds ratio.

*Adjusted for age and sex.

Then, we assessed whether genotype or allele differences of *MUC1* rs4072037 existed when patients with ASSD were stratified by the presence/absence of ILD. However, no significant differences were observed both before and after adjustment for age and sex (Table 2). It was also the case when anti Jo-1 positive ASSD patients were compared with anti Jo-1 negative ASSD ones (Table 2).

**Table 2:** Genotype and allele frequencies of *MUC1* rs4072037 in patients with ASSD stratified according to the presence/absence of ILD or anti Jo-1 antibodies.

	ASSD						ASSD					
	ILD+ % (n)	ILD− % (n)	*p*	OR [95% CI]	p*	OR [95% CI]*	Anti Jo-1+ % (n)	Anti Jo-1− % (n)	*p*	OR [95% CI]	p*	OR [95% CI]*
**Genotypes**												
TT	19.5 (29)	20.0 (3)	–	Ref.	–	Ref.	18.7 (20)	17.3 (9)	–	Ref.	–	Ref.
TC	53.0 (79)	33.3 (5)	0.52	1.63 [0.37–7.28]	0.52	1.64 [0.36–7.52]	54.2 (58)	44.2 (23)	0.79	1.13 [0.45–2.86]	0.82	1.12 [0.43–2.89]
CC	27.5 (41)	46.7 (7)	0.49	0.61 [0.14–2.54]	0.36	0.50 [0.11–2.22]	27.1 (29)	38.5 (20)	0.39	0.65 [0.25–1.72]	0.57	0.75 [0.27–2.03]
**Alleles**												
T	46.0 (137)	36.7 (11)	–	Ref.	–	Ref.	45.8 (98)	39.4 (41)	–	Ref.	–	Ref.
C	54.0 (161)	63.3 (19)	0.33	0.68 [0.31–1.48]	0.24	0.62 [0.28–1.38]	54.2 (116)	60.6 (63)	0.28	0.77 [0.48–1.24]	0.47	0.83 [0.51–1.36]

ASSD: antisynthetase syndrome; CI: confidence interval; ILD: interstitial lung disease; OR: odds ratio.

*Adjusted for age and sex.

Furthermore, we analyzed the differences in the genotype or allele frequencies of *MUC1* rs4072037 between the group of patients with ASSD-ILD+ and patients with IPF. In this regard, a statistically significant increase of *MUC1* rs4072037 TC and CC genotypes and C allele frequencies were found in ASSD-ILD+ patients when compared to those with IPF (Table 3). The same results were obtained after adjustment for age and sex (Table 3). When stratified by sex, an increase of *MUC1* rs4072037 TC and CC genotypes and C allele frequencies was disclosed in women with ASSD-ILD+ when compared to those with IPF (Supplementary table 2). In addition, higher *MUC1* rs4072037 CC genotype and C allele frequencies were observed in men with ASSD related to those with IPF (Supplementary table 3).

**Table 3:** Genotype and allele frequencies of *MUC1* rs4072037 in ASSD-ILD+ and IPF patients.

	ASSD-ILD+ % (n)	IPF% (n)	*p*	OR [95% CI]	p*	OR [95% CI]*
**Genotypes**						
TT	19.5 (29)	32.7 (57)	–	Ref.	–	Ref.
TC	53.0 (79)	48.3 (84)	**0.03**	**1.85 [1.07–3.18]**	**0.05**	**1.91 [0.99–3.68]**
CC	27.5 (41)	19.0 (33)	**0.006**	**2.44 [1.29–4.63]**	**0.007**	**2.84 [1.33–6.08]**
**Alleles**						
T	46.0 (137)	56.9 (198)	–	Ref.	–	Ref.
C	54.0 (161)	43.1 (150)	**0.006**	**1.55 [1.14–2.12]**	**0.007**	**1.68 [1.16–2.44]**

ASSD: antisynthetase syndrome; CI: confidence interval; ILD: interstitial lung disease; IPF: idiopathic pulmonary fibrosis; OR: odds ratio. Statistically significant results are highlighted in bold.

*Adjusted for age and sex.

### Serum KL-6 levels in patients with ASSD

**Figure 1:**
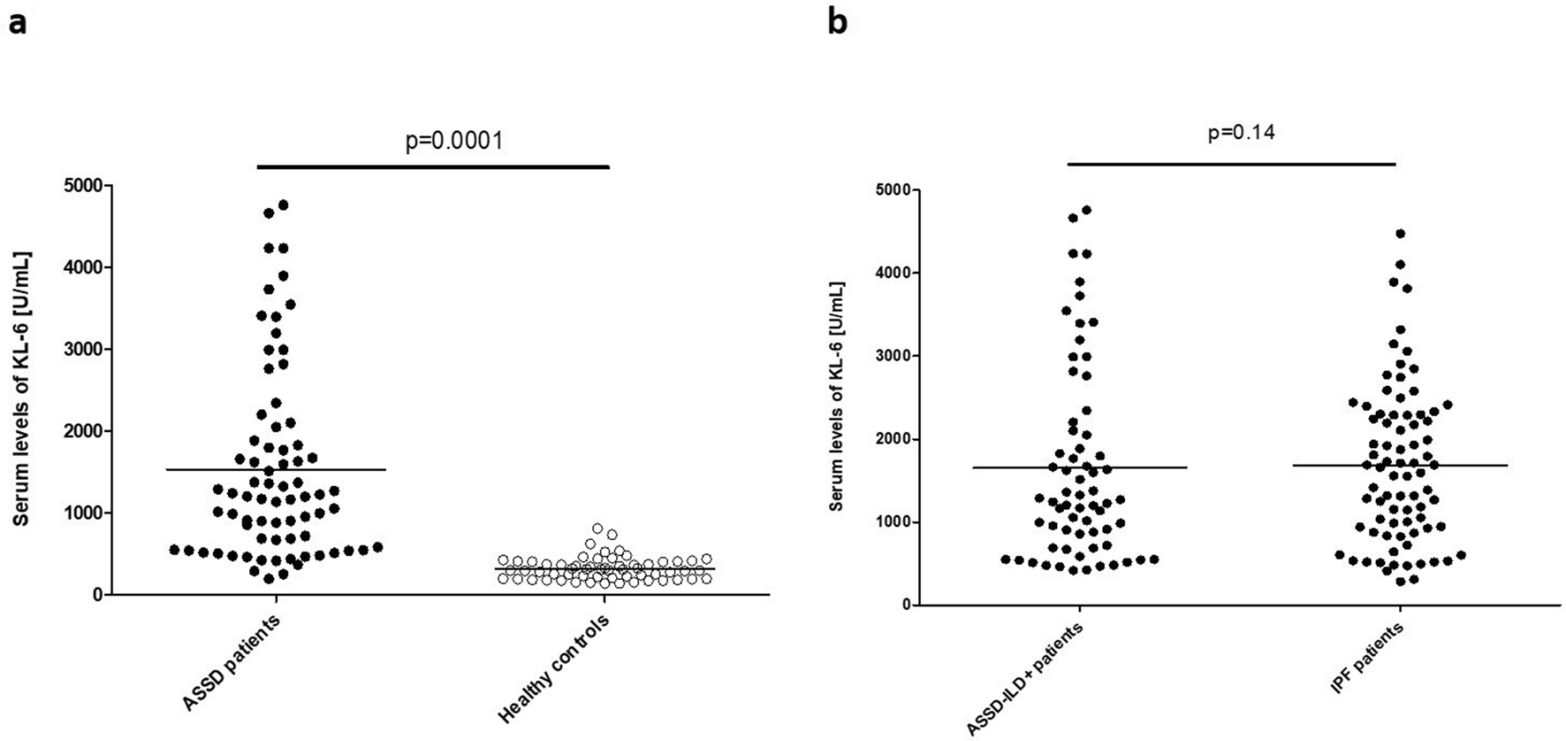
Serum KL-6 levels in patients with antisynthetase syndrome (ASSD), healthy controls and patients with idiopathic pulmonary fibrosis (IPF). (**a**) Comparison between ASSD patients and healthy controls. (**b**) Comparison between patients with ILD associated with ASSD (ASSD-ILD+) and IPF patients. P values were adjusted for age, sex, and smoking history. Horizontal bars indicate the mean value of each study group.

Next, we compared serum levels of KL-6 between the whole cohort of ASSD patients and healthy controls as well as between ASSD-ILD+ patients and IPF patients. In this sense, serum KL-6 levels were significantly higher in patients with ASSD compared to healthy controls (1533.38 ± 1159.76 U/mL *vs.* 322.39 ± 140.09 U/mL, p = 0.0001, Fig. 1a). However, there was no statistically significant difference in serum KL-6 levels between ASSD-ILD+ patients and IPF patients (1653.56 ± 1165.19 U/mL *vs.* 1682.23 ± 949.93 U/mL, p = 0.14, Fig. 1b). This was also the case when these patients were stratified by sex, being serum KL-6 levels similar between women with ASSD-ILD+ and those with IPF and also between men with ASSD-ILD+ and those with IPF (1383.20 ± 993.93 U/mL *vs.* 1561.14 ± 894.01 U/mL, p = 0.87; 2232.91 ± 1312.30 U/mL *vs*. 1707.91 ± 965.94 U/mL, p = 0.12, respectively).

### Influence of *MUC1* rs4072037 on serum KL-6 levels

**Figure 2:**
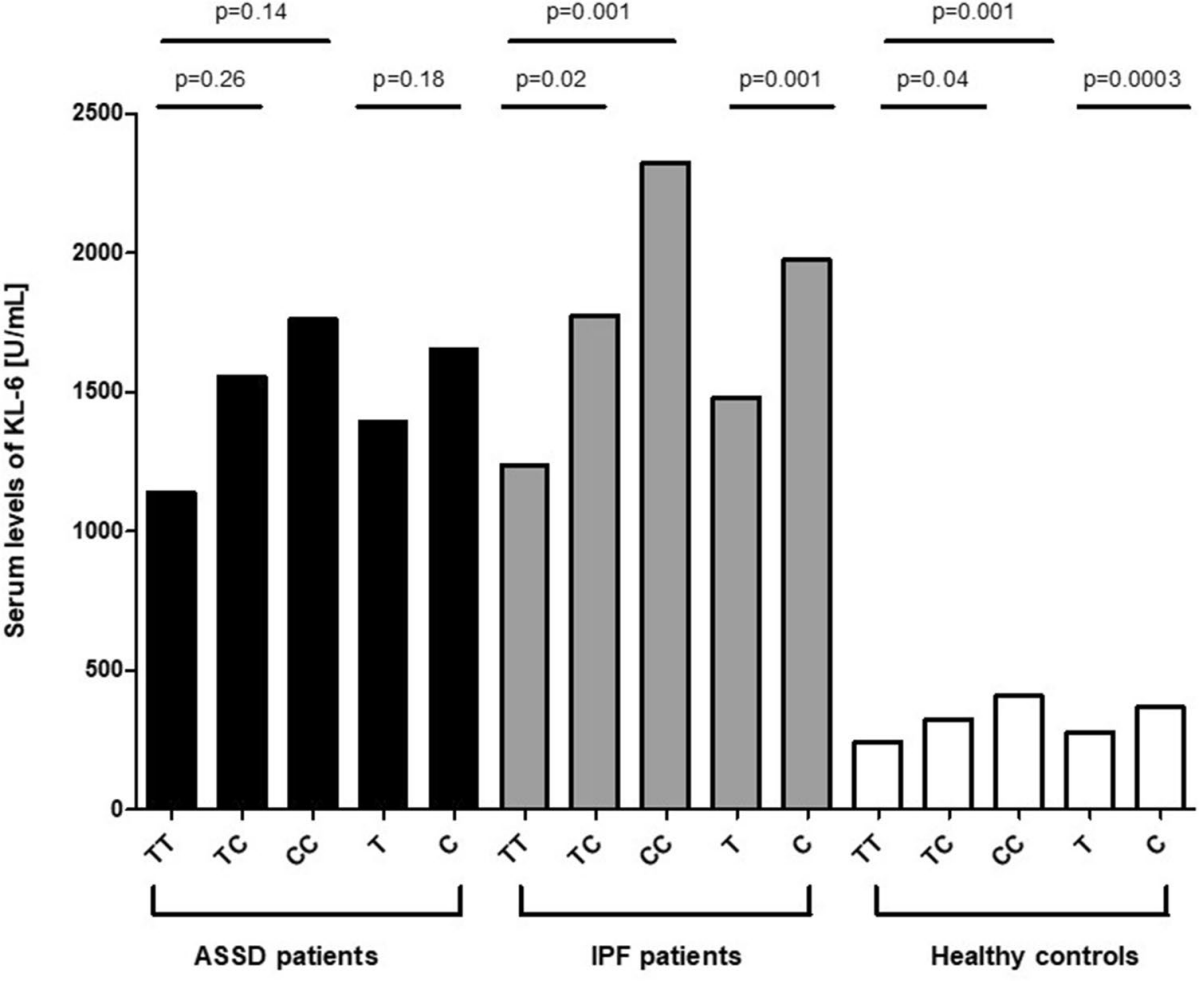
Serum KL-6 levels in patients with antisynthetase syndrome (ASSD), patients with idiopathic pulmonary fibrosis (IPF) and healthy controls, according to their genotype and allele for *MUC1* rs4072037. Bars indicate the mean value.

We also assessed the influence of *MUC1* rs4072037 genotypes and alleles on serum levels of KL-6 in ASSD patients, IPF patients and healthy controls (Fig. 2). It was interesting to note that, regardless of the study group analyzed, individuals with the *MUC1* rs4072037 CC genotype exhibited the highest serum levels of KL-6, whereas those with TC genotype had intermediate serum KL-6 levels compared to those carrying the reference TT genotype. Consequently, individuals with *MUC1* rs4072037 C allele had higher serum KL-6 levels than those carrying the reference T allele. Although these results were statistically significant in the group of patients with IPF and healthy controls, they did not reach statistical significance in ASSD patients (Fig. 2). This was also the case when we specifically analyzed ASSD-ILD+ patients.

## Discussion

The study of biomarkers associated with the susceptibility and severity to develop ASSD constitutes a field of growing interest. In this regard, mucin-1/KL-6 has been proposed as a serum biomarker useful to detect the presence of ILD, to evaluate disease activity and to predict clinical outcomes in various types of ILDs, such as IPF and CTD-ILD, including PM and DM^5,7,13,16,17^. However, after conducting a literature review, only a recent study aimed to assess serum KL-6 levels in a small cohort of Chinese ASSD patients^38^. Of note, Takei et al. suggested that changes in KL-6 levels could be a predictive factor for the recurrence of ASSD^39^. In addition, there is no information on the role of the *MUC1* rs4072037 polymorphism in ASSD patients. Accordingly, in this study we aimed to determine, for the first time, the potential implication of mucin 1 /KL-6, at the genetic and serological level, as a biomarker of ASSD susceptibility as well as for the differential diagnosis between ASSD-ILD+ and IPF patients.

In this study, we disclosed that the *MUC1* rs4072037 C allele was associated with a higher risk of developing ASSD compared to healthy controls. In addition, our results showed no association of *MUC1* rs4072037 with the presence/absence of ILD and anti Jo-1 antibody status, suggesting that its influence is unrelated to the most common clinical features of the disease. Furthermore, we found a significant increase in serum levels of KL-6 in patients with ASSD related to controls. In accordance with our results, it is well known that serum KL-6 levels are normal in healthy individuals and elevated in other CTDs^7,9,25,27,28^.

Our study also revealed that *MUC1* rs4072037 C allele has a different distribution between ASSD-ILD+ and IPF patients while serum KL-6 levels were similar between both groups, regardless of the sex of the patients. These results may appear to be controversial at first glance. However, it is plausible to think that the different genetic distribution of rs4072037 found between ASSD-ILD+ and IPF patients is not reflected in their serum KL-6 levels due to molecular regulations that may occur at the epigenome, transcriptome or post-transcriptional level. In this regard, it should also be noted that, in keeping with our results regarding KL-6 levels, there was no significant difference in serum KL-6 levels between 43 ASSD-ILD patients and 34 IPF patients from China^38^. In addition, Ishii et al. reported that KL-6 serum levels cannot be used to distinguish patients with IPF from those with non-specific interstitial pneumonia^40^. Taking all this into account, although the measurement of KL-6 allows us to differentiate a healthy subject from an ILD patient, our results indicate that it would not be useful for the discrimination between ASDD-ILD + and IPF patients. On the contrary, our genetic results support the use of *MUC1* rs4072037 genotyping as a diagnostic tool to identify patients with ILD who do not meet criteria for IPF and that, in case of having the *MUC1* rs4072037 C allele, can have a greater risk of presenting ASSD. In this sense, this genetic biomarker may help clinicians to generate a higher level of suspicion towards the diagnosis of ASSD, irrespective of their serum levels of KL-6.

In line with the above, we previously reported that a polymorphism in *MUC5B* gene, that codifies for another relevant lung mucin, showed genetic differences between ASSD-ILD+ and IPF^31^. Taken together, these findings suggest that mucin genetic polymorphisms may influence the phenotype expression of diseases associated with ILD. This is crucial given the diverse prognosis and therapeutic options of each disease. Given that the frequency of ASSD patients seen in ILD units is high^41^, these genetic biomarkers can constitute an affordable and easy-to-perform complementary tool to contribute to the early recognition and management of this entity.

Finally, in our study we found an association between *MUC1* rs4072037 C allele and higher serum KL-6 levels. This finding was in line with previous reports that included healthy controls and patients with diverse lung diseases^9,14,18^.

In conclusion, our results suggest that the presence of the *MUC1* rs4072037 C allele increases the risk of ASSD. It could be used as a genetic biomarker for the differential diagnosis between patients with ASSD-ILD+ and IPF. In addition, KL-6 can be a good marker of ILD regardless of the underlying condition.

## Supplementary Information

Supplementary Tables.

## Data Availability

All data generated or analyzed during this study are included in this published article.
