# Supplementary material for: Role of MUC1 rs4072037 polymorphism and serum KL-6 levels in patients with antisynthetase syndrome
Source: Sci Rep. 2021 Nov 19;11:22574. doi: 10.1038/s41598-021-01992-y (PMC8604941; doi:10.1038/s41598-021-01992-y)
Supplement: Supplementary file 1 — Supplementary Tables. [file 41598_2021_1992_MOESM1_ESM.pdf]

Supplementary table 1. Main demographic and baseline lung function information of the ASSD-ILD+ and IPF patients included in this study. ASSD: antisynthetase syndrome; DLCO: diffusing capacity of the lung for carbon monoxide; FVC: forced vital capacity; ILD: interstitial lung disease; IPF: idiopathic pulmonary fibrosis; SD: standard deviation.

	ASSD-ILD+ patients	IPF patients
Age at the time of the study (years), mean ± SD	59.1 ± 13.9	64.4 ± 7.1
Sex (men/women), n (%)	48/98 (32.9 / 67.1)	144/30 (82.8 / 17.2)
Smoking history, n (%)	54 (42.5)	139 (81.8)
Lung function		
FVC (% predicted), mean ± SD	77.6 ± 25.3	74.5 ± 21.0
DLCO (% predicted), mean ± SD	58.7 ± 22.6	35.1 ± 15.6

Supplementary table 2. Genotype and allele frequencies of *MUC1* rs4072037 in women with ASSD-ILD+ and IPF. ASSD: antisynthetase syndrome; CI: confidence interval; ILD: interstitial lung disease; IPF: idiopathic pulmonary fibrosis; OR: odds ratio. Statistically significant results are highlighted in **bold**.

	ASSD-ILD+<br>% (n)	IPF<br>% (n)	*p*	OR [95% CI]	*p**	OR [95% CI]*
Genotypes						
TT	18.4 (18)	40.0 (12)	-	Ref.	-	Ref.
TC	57.1 (56)	43.3 (13)	**0.03**	**2.87 [1.11-7.41]**	**0.04**	**2.89 [1.06-7.89]**
CC	24.5 (24)	16.7 (5)	0.06	3.20 [0.96-10.72]	**0.04**	**3.72 [1.04-13.29]**
Alleles						
T	46.9 (92)	61.7 (37)	-	Ref.	-	Ref.
C	53.1 (104)	38.3 (23)	**0.05**	**1.82 [1.01-3.28]**	**0.03**	**1.96 [1.05-3.64]**

*Adjusted for age.

Supplementary table 3. Genotype and allele frequencies of *MUC1* rs4072037 in men with ASSD-ILD+ and IPF. ASSD: antisynthetase syndrome; CI: confidence interval; ILD: interstitial lung disease; IPF: idiopathic pulmonary fibrosis; OR: odds ratio. Statistically significant results are highlighted in **bold**.

	ASSD-ILD+ % (n)	IPF % (n)	*p*	OR [95% CI]	*p**	OR [95% CI]*
Genotypes						
TT	20.8 (10)	31.3 (45)	-	Ref.	-	Ref.
TC	45.9 (22)	49.3 (71)	0.44	1.39 [0.60-3.22]	0.43	1.41 [0.61-3.26]
CC	33.3 (16)	19.4 (28)	**0.04**	**2.57 [1.02-6.45]**	0.07	2.40 [0.95-6.08]
Alleles						
T	43.8 (42)	55.9 (161)	-	Ref.	-	Ref.
C	56.2 (54)	44.1 (127)	**0.04**	**1.63 [1.02-2.60]**	0.06	1.57 [0.98-2.52]

*Adjusted for age.
